# Hypoxia following etorphine administration in goats (*Capra hircus*) results more from pulmonary hypertension than from hypoventilation

**DOI:** 10.1186/s12917-015-0337-5

**Published:** 2015-02-03

**Authors:** Leith Carl Rodney Meyer, Robyn Sheila Hetem, Duncan Mitchell, Andrea Fuller

**Affiliations:** Department of Paraclinical Sciences, Faculty of Veterinary Science, University of Pretoria, Onderstepoort, 0110 South Africa; Brain Function Research Group, School of Physiology, University of the Witwatersrand, 7 York Road, Parktown, 2193 South Africa

**Keywords:** Hypoxia, Opioid, Respiratory depression, Oxygen diffusion

## Abstract

**Background:**

Etorphine, a potent opioid agonist, causes pulmonary hypertension and respiratory depression. Whether etorphine-induced pulmonary hypertension negatively influences pulmonary gas exchange and exacerbates the effects of ventilator depression and the resultant hypoxemia is unknown. To determine if these effects occurred we instrumented twelve goats with peripheral and pulmonary arterial catheters to measure systemic and pulmonary pressures before and after etorphine administration. Concurrent cardiopulmonary and arterial blood gas variables were also measured.

**Results:**

Etorphine induced hypoventilation (55% reduction to 7.6 ± 2.7 L.min^−1^, F_(11,44)_ = 15.2 *P* < 0.0001), hypoxia (<45 mmHg, F_(11,44)_ = 8.6 *P* < 0.0001), hypercapnia (>40 mmHg, F_(11,44)_ = 5.6 *P* < 0.0001) and pulmonary hypertension (mean 23 ± 6 mmHg, F_(11,44)_ = 8.2 *P* < 0.0001). Within 6 min of etorphine administration hypoxia was twice (F_(11,22)_ = 3.0 *P* < 0.05) as poor than that expected from etorphine-induced hypoventilation alone. This disparity appeared to result from a decrease in the movement of oxygen (gas exchange) across the alveoli membrane, as revealed by an increase in the P(A-a)O_2_ gradient (F_(11,44)_ = 7.9 *P* < 0.0001). The P(A-a)O_2_ gradient was not correlated with global changes in the ventilation perfusion ratio (P = 0.28) but was correlated positively with the mean pulmonary artery pressure (P = 0.017, r^2^ = 0.97), indicating that pulmonary pressure played a significant role in altering pulmonary gas exchange.

**Conclusion:**

Attempts to alleviate etorphine-induced hypoxia therefore should focus not only on reversing the opioid-induced respiratory depression, but also on improving gas exchange by preventing etorphine-induced pulmonary hypertension.

## Background

Although opioids are effective in reducing pain and inducing anaesthesia, sedation or chemical immobilization, their use is not without complications or harmful side-effects. Of these side-effects, respiratory compromise is the most detrimental and well known [1-3]. Patients given opioids may develop hypercapnia, respiratory acidosis and hypoxia [4,5]. The respiratory compromise usually is attributed to respiratory depression, characterised by slowing of breathing frequency and decreased respiratory responses to hypercapnia, caused by the depressive effects of the opioids on the respiratory network and other neurons in the brain-stem [6,7]. Opioids also depress hypoxic and hypercapnic ventilator responses by acting on peripheral chemoreceptors [6,8].

We have reason to believe, though, that respiratory depression is not the sole cause of opioid-induced hypoxia. Serotonergics drugs improve respiratory rhythmogenesis through their effects on respiratory neurons [3,5,8]. However, when we used serotonergic drugs, acting on 5-HT1A and 5-HT4 receptors, to reverse etorphine-induced respiratory compromise, we found that their hypoxia-relieving effects were not related solely to their actions on respiratory neurons, but also their effects on pulmonary gas exchange [4,9]. We postulated that the improvements in gas exchange were brought about by vasodilatory effects of these serotonergic drugs, which countered etorphine’s vasoconstrictor effects in the pulmonary circulation [7].

We now have tested that postulate. We determined how etorphine, a potent opioid agonist widely used in chemical immobilization of wildlife [2], altered pulmonary arterial blood pressure and gas exchange, and established how much these effects contributed to etorphine-induced hypoxia. We measured blood gases, pulmonary pressures and other cardiorespiratory variables before and after etorphine administration in goats.

## Methods

Twelve adult female mix breed goats (*Capra hircus*), weighing 33 ± 5 kg (mean ± SD), were used in this mechanistic study. They were housed together in temperature-controlled indoor pens (~22°C) in Johannesburg, at an altitude of 1753 m, on a 12 hour light/dark cycle (lights on 6:00). The goats were reared at this altitude and therefore adapted to lower inspired partial pressures of oxygen than at sea-level. They had water *ad libitum* and were fed on hay and sheep concentrate pellets. For six weeks before the start of data collection the goats were habituated to being restrained in sternal recumbency on a work table with a face mask over their muzzle, for a 5 min period every other day. The procedures were approved by the University of the Witwatersrand’s Animal Ethics Screening Committee (clearance number 2008/49/04).

Goats were weighed 48 hours before, and starved for 24 hours before etorphine administration to reduce the risk of bloating and of regurgitation of ingesta. On the day of etorphine administration, each goat was restrained in sternal recumbency and its ears and left lateral neck area was shaved and swabbed with 5% chlorhexidine gluconate (Hibitane, Astra Zeneca, Johannesburg, South Africa) in 100% ethanol. A 22 gauge intravenous catheter (Introcan, B/Braun, Melsungen, Germany) was placed in one of the auricular arteries and connected via a fluid-filled arterial line to a pre-calibrated Deltran II pressure transducer (DPT-200, Utah Medical Products, Midvale, U.S.A.). Local anaesthetic (2 ml lignocaine, Bayer, South Africa) was injected subcutaneously around the left jugular vein before an 2.8 mm (outside diameter) introducer sheath (1350BF85, Edwards Life Sciences, Irene, South Africa) was inserted into the vein. A fluid-filled Swan-Ganz Catheter (Continuous Cardiac Output Thermodilution Catheter, 139HF75P, Edwards Life Sciences, Irene, South Africa), which was connected to another Deltran II pressure transducer, was inserted through this sheath and advanced through the vein and right heart. Under the guidance of the real-time pressure trace, the tip of the catheter was positioned in the pulmonary artery such that when the balloon of the catheter was inflated it “wedged” the artery. This placement enabled the measurement of both pulmonary artery (averaged over 40s every minute) and pulmonary artery wedge (averaged over 10s every second minute) pressures. Through a three-way stopcock, a separate side-port of the Swan-Ganz catheter was connected to the pressure transducer to measure central venous pressure (averaged over 10s every other second minute). To standardise pressure readings the pressure transducers were placed at the level of the scapulohumeral joint (level of the heart base). The Swan-Ganz transducers were connected via blood pressure amplifiers (FE117, ADIntruments, Castle Hill, Australia) to a PowerLab Exercise Physiology System (ML870B80, ADIntruments, Castle Hill, Australia), which captured and displayed real-time data through LabChart software (Chart 5, ADIntruments, Castle Hill, Australia). The Swan-Ganz catheter also was connected to a Vigilance Monitor (Edwards Life Sciences, Irene, South Africa), which continuously measured pulmonary artery temperature and cardiac output based on thermodilution principles. Pulmonary artery temperature was used as the body temperature needed to calculate water vapour pressure in alveolar air and to allow for temperature-corrected blood gas measurements.

A clear canine anaesthetic face mask (J-298C, Jorgensen Laboratories, Loveland, USA) was placed over the muzzle of the goat and positioned so as to limit dead space. A gasket made from a latex glove formed a tight seal between the goat’s muzzle and the face mask. The face mask was connected to a two-way valve which directed all the expired air into the PowerLab Exercise Physiology System, via a respiratory flow head (MLT1000L) linked to a spirometer (ML140) and a gas mixing chamber (MLA245), in which expired gas temperature was measured by a thermistor pod (ML309). The data from these modules were collected via the PowerLab 8/30 amplifier (ML870) and integrated with the Metabolic Module software to measure (at BTPS - body temperature and pressure saturated) minute ventilation (L.min^−1^) and respiratory rate. Prior to each set of measurements the spirometer was calibrated using a 3 L calibration syringe.

Data were recorded from 4 min before the injection of etorphine hydrochloride (Captivon, Wildlife Pharmaceuticals, Whiteriver, South Africa) until 15 min after the injection. Etorphine was injected intramuscularly into the gluteus muscles using a 20G hypodermic needle at a dose of 0.1 mg.kg^−1^ (the drug was diluted in injectable water to standardise the volume injected to 1 ml) a dose shown in pilot studies to induce motionless immobilization for the 15 min of data recording and to have no long-term sequelae. Once immobile, the goats were positioned by a handler holding the horns so that the neck was aligned with the spinal column and the head was elevated above the thorax with the nose pointing downwards. This positioning allowed for unobstructed eructation of ruminal gas and an open upper airway. A 0.5 ml auricular arterial blood sample was drawn 2 min before, and at 2, 6, 10 and 14 min after etorphine injection (these time intervals were selected *a priori*); the catheter was flushed with 2 ml heparinized saline (5 iu.ml^−1^, Heparin, Intramed, Johannesburg, South Africa) after each sample. Directly after each sample was drawn, the arterial partial pressure of oxygen (PaO_2_), carbon dioxide (PaCO_2_) and pH were measured on a pre-calibrated blood gas analyzer with pre-calibrated blood gas cassettes (Roche OPTI CCA Analyzer + OPTI cassette B, Kat Medical, Johannesburg, South Africa); data were reported at 37°C.

At the end of the recording period, all instruments were removed and the goats were returned to their pens, where the action of etorphine was antagonised by 2 mg.kg^−1^ naltrexone hydrochloride (Trexinol, Wildlife Pharmaceuticals, White River, South Africa) injected intramuscularly.

All measurements were made indoors, between 2 hours and 10 hours after lights-on, at an ambient dry-bulb temperature between 20°C and 22°C and relative humidity between 21% and 24%. Barometric pressures were measured to an accuracy of 0.1 mmHg, by the on-board barometer of the blood gas analyzer, which had been calibrated against a Fortin mercury barometer (On, F.D & Co Ltd, United Kingdom). Barometric pressure ranged from 628 mmHg to 634 mmHg. At that barometric pressure, the partial pressure of inspired oxygen was about 133 mmHg.

We used GraphPad Prism version 6 for Windows (GraphPad Software, San Diego, USA) for statistical analyses. All results are reported as mean ± SD, and a *P* < 0.05 was considered statistically significant. Partial pressure of alveolar oxygen (PAO_2_) was determined by using the Alveolar Gas Equation (FiO_2_ (Pb – PH_2_O) – PaCO_2_/RQ) where FiO_2_ is the fractional inspired oxygen (0.209), Pb the measured barometric pressure (mmHg) and PH_2_O the water vapour pressure of saturated air in the alveoli. PH_2_O (mmHg) was calculated as 4.58 e{(17.27 × Tb)/(237.3 + Tb)} [10], where Tb is the body temperature. We assumed that the partial pressure of CO_2_ in the alveoli was equal to the arterial partial pressure of CO_2_ (PaCO_2_) and used a RQ (respiratory quotient) value of 1, the norm in conscious healthy goats [11]. The alveolar-arterial oxygen partial pressure gradient P(A-a)O_2_ was calculated as PAO_2_ - PaO_2_. Expected-PaO_2_, a theoretical value of arterial partial pressure of oxygen that would be expected if the PaO_2_ after etorphine administration was determined only by PAO_2_, which would decrease when ventilation decreased, was calculated by assuming that P(A-a)O_2_ remained at the value attained immediately before etorphine administration. Pulmonary vascular resistance was calculated by dividing the difference between the pulmonary artery and wedge pressure by the cardiac output. Global ventilation perfusion ratios were calculated by dividing ventilation by cardiac output.

A repeated measures two-way ANOVA followed by a Šídák’s post-hoc test for multiple comparisons was used to test for differences between measured PaO_2_ and expected PaO_2_ across time following etorphine administration. A repeated measures one-way ANOVA followed by Dunnett’s post-hoc test for multiple comparisons with a control was used to test for differences between values 2 min before etorphine administration and those after etorphine administration, for respiration rate, ventilation, heart rate, cardiac output, ventilation perfusion ratio, PaCO_2_, P(A-a)O_2_, systemic arterial pressures, pulmonary arterial pressures, pulmonary arterial wedge pressure and pulmonary vascular resistance at each time point that blood gases were measured (2, 6, 10 and 14 min) after etorphine administration. Pearson’s correlations and linear regressions were used to test for linear relationships between mean (averaged over 12 animals) P(A-a)O_2_, PaO_2_, mean pulmonary artery pressure and other cardiorespiratory variables. Although the correlations were similar when pre-etorphine data were included, only post-etorphine administration data were included to avoid cloud regressions.

## Results

### Effects of etorphine on immobilization and cardiorespiratory function

**Figure 1:**
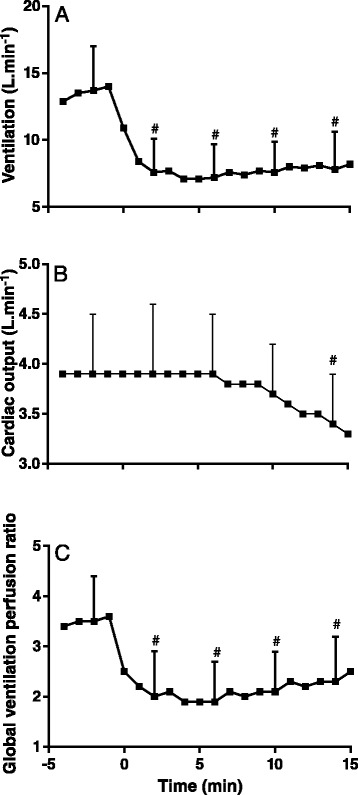
**Effects of etorphine (0.1 mg.kg** ^**−1**^ **) injected intramuscularly (time = 0) on ventilation (A), cardiac output (B) and global ventilation perfusion ratio (C) in goats (mean ± SD, n = 12).** # *P* < 0.05 pre-etorphine vs. post-etorphine (repeated measures one-way ANOVA with post hoc Dunnett’s test) at time 2, 6, 10 and 14 min.

Administration of etorphine induced immobilization within 2–3 min, following which the goats remained motionless and unresponsive to moderate sound (talking) and physical stimuli (blood sampling and the palpebral reflex test) for the 15 min of measurements. Before etorphine administration (−2 min) the goats had a respiratory rate of 35 ± 10 breaths.min^−1^ and minute ventilation was 13.7 ± 3.3 L.min^−1^. Following etorphine administration, both respiratory rate (F_(11,44)_ = 11.9 *P* < 0.0001) and ventilation (F_(11,44)_ = 15.2 *P* < 0.0001) decreased significantly. Within 2 min of etorphine administration the respiratory rate decreased to 16 ± 7 breaths.min^−1^ and minute ventilation to 7.6 ± 2.7 L.min^−1^ (Figure 1A), levels close to which they remained throughout the immobilization. Heart rate, which was 108 ± 25 beats.min^−1^ before etorphine administration, did not change significantly following etorphine administration (F_(11,44)_ = 4.1 *P* = 0.30). Cardiac output (3.9 ± 0.7 L.min^−1^ before etorphine administration) did not change immediately after etorphine administration, but decreased gradually from 3.8 ± 0.5 L.min^−1^ at 6 min to 3.4 ± 0.5 L.min^−1^ at the end of the immobilization (14 min), resulting in cardiac output being significantly lower overall, following etorphine administration (F_(11,44)_ = 18.9 *P* = 0.01, Figure 1B). Global ventilation perfusion ratio across the lung, which was 3.5 ± 0.9 before etorphine administration, decreased to 2.0 ± 0.9 at 2 min, and remained low (2.1 ± 0.8) throughout the immobilization, so that, overall, the ratio was significantly lower following etorphine administration (F_(11,44)_ = 32.3 *P* < 0.0001, Figure 1C).

### Effects of etorphine on blood gases

**Figure 2:**
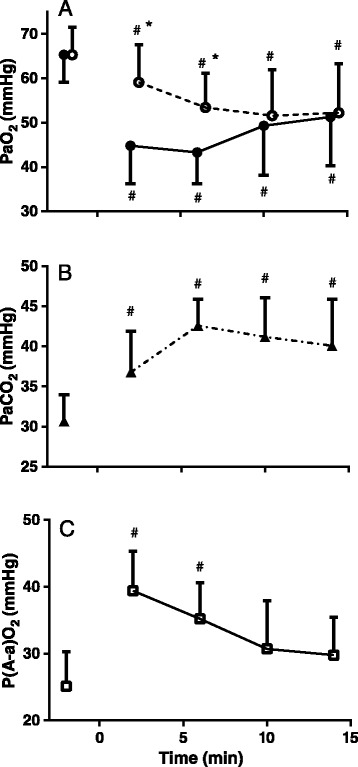
**Effects of etorphine (0.1 mg.kg** ^**−1**^ **) injected intramuscularly (time = 0) on measured arterial partial pressure of oxygen (PaO**_**2**_ **, solid symbols) and the theoretical value of arterial partial pressure of oxygen calculated assuming that alveolar-arterial oxygen partial pressure gradient P(A-a)O**_**2**_ **remained constant (Expected-PaO**_**2**_ **, open symbols) (A), arterial partial pressure of carbon dioxide (B) and alveolar-arterial oxygen partial pressure gradient P(A-a)O**_**2**_ **(C) in goats (mean ± SD, n = 12).** # *P* < 0.05 pre-etorphine vs. post-etorphine (repeated measures two-way ANOVA with post hoc Sidak’s multiple comparison test **(A)** and repeated measures one-way ANOVA with post hoc Dunnett’s test **(B&C)**), * *P* < 0.05 PaO_2_ vs. expected-PaO_2_ (repeated measures two-way ANOVA with post hoc Sidak’s multiple comparison test **(A)**) at time 2, 6, 10 and 14 min.

Figure 2A shows the effect of etorphine on measured arterial partial pressure of oxygen (PaO_2_). PaO_2_ was 65 ± 6 mmHg before etorphine administration (−2 min) and decreased to below 45 mmHg at 2 and 6 min after etorphine administration (F_(11,44)_ = 8.6 *P* < 0.0001), before gradually increasing again. The arterial partial pressure of carbon dioxide (PaCO_2_) before etorphine administration was 31 ± 3 mmHg; after etorphine administration it increased (F_(11,44)_ = 5.6 *P* < 0.0001) to above 40 mmHg (9–12 mmHg increase) from 6 min to the end of the immobilization (Figure 2B). The alveolar arterial oxygen partial pressure difference (P(A-a)O_2_) was 25 ± 5 mmHg before etorphine administration and increased after etorphine administration (F_(11,44)_ = 7.9 *P* < 0.0001), although it had returned to the pre-etorphine difference within 10 min after etorphine administration (Figure 2C).

Following etorphine administration, the calculated arterial partial pressure of oxygen that would be expected if the partial pressure of oxygen was influenced only by a decrease in ventilation, that is assuming that P(A-a)O_2_ did not change, was lower than before etorphine administration (F_(11,44)_ = 22.7 *P* < 0.0001), and was greater than measured PaO_2_ (F_(11,22)_ = 3.0 *P* < 0.05), though expected and measured values were the same after 10 min after etorphine administration (Figure 2A).

### Effects of etorphine on the systemic and pulmonary vasculature

**Figure 3:**
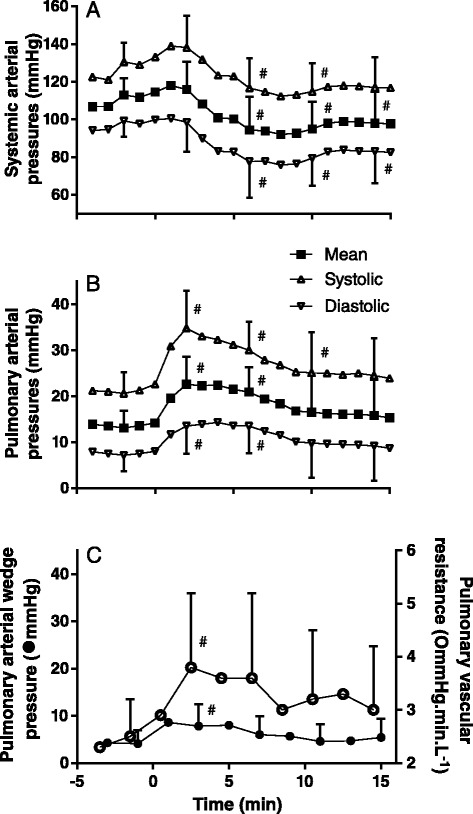
**Effects of etorphine (0.1 mg.kg** ^**−1**^ **) injected intramuscularly (time = 0) on systemic artery (A), pulmonary artery (B) and pulmonary artery wedge pressures and pulmonary vascular resistance (C) in goats (mean ± SD, n = 12).** # *P* < 0.05 pre-etorphine vs post-etorphine (one-way ANOVA with post hoc Dunnett’s test) at time 2, 6, 10 and 14 min.

After etorphine administration, the systemic arterial pressures decreased (systolic F_(11,44)_ = 6.8 *P* < 0.0001; mean F_(11,44)_ = 8.2 *P* < 0.0001; diastolic F_(11,44)_ = 8.6 *P* < 0.0001), with decreases significant after 6 min (Figure 3A). Before the administration of etorphine (−2 min), pulmonary artery systolic pressure was 21 ± 5 mmHg, mean was 13 ± 4 mmHg and diastolic was 7 ± 4 mmHg (Figure 3B). Pulmonary artery pressures increased after etorphine administration (systolic F_(11,44)_ = 6.8 *P* < 0.0001, mean F_(11,44)_ = 8.2 *P* < 0.0001, diastolic F_(11,44)_ = 8.6 *P* < 0.0001), with systolic, mean and diastolic pressures all being significantly higher within 2 min after etorphine administration but returning to pre-etorphine values by the end of the immobilization. Pulmonary vascular resistance (F_(11,44)_ = 2.6 *P* = 0.01) and pulmonary artery wedge pressure (F_(11,44)_ = 11.88 *P* < 0.0001) were increased after etorphine administration from 2.9 ± 1.1 mmHg.min.L^−1^ and from 4.1 ± 2.9 mmHg respectively (Figure 3C), but only because values 3 min after etorphine administration were elevated significantly.

### Factors influencing arterial partial pressure of oxygen

**Figure 4:**
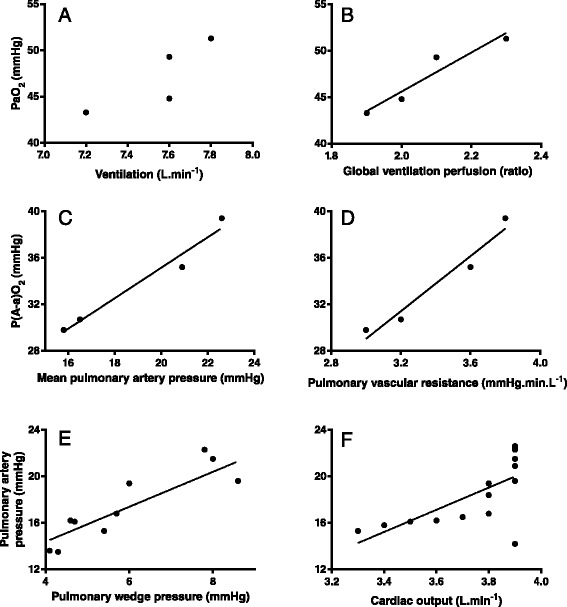
**Mean arterial partial pressure of oxygen (PaO2)** ***vs.*** **mean ventilation (A,** ***r*** ^**2**^ **= 0.7,** ***P*** **= 0.16), mean arterial partial pressure of oxygen (PaO2)** ***vs.*** **mean global ventilation perfusion ratio (B,** ***r*** ^**2**^ **= 0.92,** ***P*** **= 0.04,** ***y*** **= 21** ***x*** **+ 3.6), mean alveolar-arterial oxygen partial pressure gradient** ***vs.*** **mean of mean pulmonary artery pressure (C,** ***r*** ^**2**^ **= 0.97,** ***P*** **= 0.017,** ***y*** **= 1.3** ***x*** **+ 8.9), mean alveolar-arterial oxygen partial pressure gradient** ***vs.*** **mean pulmonary vascular resistance (D,** ***r*** ^**2**^ **= 0.95,** ***P*** **= 0.024,** ***y*** **= 11.9** ***x*** **– 6.5), mean pulmonary artery pressure** ***vs.*** **mean pulmonary artery wedge pressure (E,** ***r*** ^**2**^ **= 0.77,** ***P*** **= 0.004,** ***y*** **= 1.5** ***x*** **+ 8.4), mean pulmonary artery pressure** ***vs.*** **cardiac output (F,** ***r*** ^**2**^ **= 0.47,** ***P*** **= 0.003,** ***y*** **= 9.5** ***x*** **– 17.2).** Data were averaged for 12 goats and *r* is Pearson’s correlation coefficient.

Although etorphine administration resulted in reduced ventilation and PaO_2_, after etorphine administration there was no significant linear relationship between ventilation and the PaO_2_ (Figure 4A, P = 0.16, r^2^ = 0.70). In support of the poor influence of ventilation on PaO_2_ after etorphine administration, we found that the arterial partial pressure of carbon dioxide (PaCO_2_, P = 0.98, r^2^ < 0.01) and the calculated alveolar partial pressure of oxygen (PAO_2_, P = 0.99, r^2^ < 0.01), both variables associated with the magnitude of ventilation, were not correlated with PaO_2_. Global pulmonary perfusion, measured as cardiac output (P = 0.60, r^2^ = 0.88), also was not correlated to PaO_2_. However, the relationship between global ventilation and perfusion (the ventilation perfusion ratio) after etorphine administration was correlated with the PaO_2_ (P = 0.04, r^2^ = 0.92, Figure 4B). Therefore, the changes in the PaO_2_ appeared to be attributed mainly to intrapulmonary ventilation-perfusion mismatching rather than the pump action of breathing i.e. ventilation. That the main problem lay with altered oxygen movement across the alveoli membrane, not oxygen delivery, was confirmed by the large increase in the alveolar-arterial oxygen partial pressure gradient (P(A-a)O_2_) soon after etorphine administration (Figure 2C). After etorphine administration, P(A-a)O_2_ was linearly correlated with mean pulmonary artery pressure (Figure 4C, P = 0.017, r^2^ = 0.97) and pulmonary vascular resistance (Figure 4D, P = 0.023, r^2^ = 0.95), but not significantly correlated with global ventilation (P = 0.59, r^2^ = 0.17), cardiac output (P = 0.17, r^2^ = 0.69), global ventilation perfusion ratio (P = 0.28, r^2^ = 0.52) or pressures in the left atrium as indicated by pulmonary artery wedge pressure (P = 0.07, r^2^ = 0.87).

Pulmonary artery pressure after etorphine administration was correlated with pulmonary vascular resistance (P = 0.04, r^2^ = 0.52), pulmonary artery wedge pressure (Figure 4E, P = 0.0004, r^2^ = 0.77) and cardiac output (Figure 4F, P = 0.003, r^2^ = 0.47), but not PAO_2_ (P = 0.61, r^2^ = 0.15). PAO_2_ was also not correlated with pulmonary vascular resistance (P = 0.28, r^2^ = 0.51).

## Discussion

Intramuscular administration of 0.1 mg.kg^−1^ etorphine, a highly-potent opioid receptor agonist, induced rapid chemical immobilization and decreased the partial pressure of oxygen in arterial blood (Figure 2A). One cause of this exacerbated hypoxia could be hypoventilation, which opioid agonists are well known to induce (4–6). Our goats did develop hypoventilation (Figure 1A) with the expected concomitant hypercapnia (Figure 2B), but hypoventilation was not the only, nor even the primary, cause of the hypoxia. In the first six minutes after etorphine administration, when the hypoxia was most severe, the arterial partial pressure of oxygen was about half that which would have resulted from hypoventilation alone (Figure 2A). So not only was oxygen delivery to the alveoli compromised but so to was exchange of oxygen between alveoli and arterial blood. The alveolar-arterial oxygen partial pressure gradient (P(A-a)O_2_) increased precipitously within 2 min of etorphine administration (Figure 2C). We identified one direct cause of the compromise of alveolar-arterial oxygen exchange: immediately after etorphine administration pulmonary vascular resistance also increased precipitously (Figure 3C) and pulmonary hypertension developed (Figure 3B). The alveolar-arterial oxygen partial pressure gradient (P(A-a)O_2_) after etorphine administration was correlated strongly with mean pulmonary artery pressure (Figure 4C) indicating that the pulmonary hypertension likely played a major role in hindering oxygen movement (gas exchange) across the alveoli membrane. It is reasonable to speculate that hypoxia could have been brought about by hydrostatic fluid shifts causing oedema of the interstitium, and possibly also the alveoli, so increasing diffusion distance and obstructing oxygen transfer into the blood.

Pulmonary hypertension can develop without pulmonary vascular resistance increasing, for example if global pulmonary blood flow increases. In our goats, cardiac output, and therefore global pulmonary blood flow, had not changed at the time at which the hypoxia was most severe (Figure 1B). Although cardiac output was correlated with pulmonary artery pressures (Figure 4F) this relationship was poor and resulted mainly from the decrease in cardiac output and pulmonary artery pressures towards the end of the immobilization. Another possible cause of pulmonary hypertension is increase in left atrial pressure. In our goats, pulmonary artery wedge pressure, the surrogate for left atrial pressure, did increase following etorphine administration (Figure 3C) and mean pulmonary artery pressures did correlate positively with pulmonary artery wedge pressures (Figure 4E), so increases in pressure in the left atrium could have contributed to the pulmonary hypertension. However, changes in pulmonary artery wedge pressure could not have entirely accounted for the almost two-fold increase in the mean pulmonary artery pressure, and we believe that the main contributor to the pulmonary hypertension must have been the increased pulmonary vascular resistance.

The most-likely cause of the increased pulmonary vascular resistance was pulmonary vasoconstriction. Pulmonary vasoconstriction following etorphine administration may result from activation of the sympathetic nervous system by etorphine [12], from the indirect effects of the hypoxia and hypercapnia [13] induced by etorphine [14,15], or by other unknown actions of etorphine on the pulmonary vasculature. However, generalised sympathetic activation appeared not to occur in our goats, as heart rate and systemic artery pressure did not increase. Hypoxic pulmonary vasoconstriction [16], brought about by etorphine-induced hypoventilation, may have contributed to the increased pulmonary artery pressures, but it did not appear to be the predominant factor, as changes in ventilation, as indicated by the partial pressures of alveolar oxygen and arterial carbon dioxide, were not correlated with the changes in pulmonary artery pressures and the partial pressure of alveolar oxygen was not correlated to pulmonary vascular resistance. The precise mechanisms underlying etorphine-induced and other possible opioid-induced pulmonary vasoconstriction still need to be elucidated.

If, indeed, etorphine administration induced pulmonary vasoconstriction, there is another possible contributor to the impaired oxygen transfer between alveoli and arterial blood, in addition to the impairment resulting from pulmonary hypertension. Less oxygen may have entered the arterial blood because there was less time for gas exchange to occur [16,17] after etorphine administration. Because pulmonary blood flow (cardiac output, Figure 1B) had not changed at the time at which pulmonary vascular resistance (Figure 3C) and, we presume, vasoconstriction were greatest, the speed of red cells traversing the narrowed pulmonary vessels would have increased. If arterial blood traversed the pulmonary capillary in less than 0.25 seconds, oxygen would not have enough time to diffuse and equilibrate in the blood [16].

Although we believe that pulmonary vasoconstriction and the resultant pulmonary hypertension were the main factors impairing oxygen exchange between alveoli and arterial blood, other factors may have been operating too. Following etorphine administration, there was a strong relationship between the PaO_2_ and the global ventilation perfusion ratio (Figure 4B), but there was no significant relationship between global ventilation perfusion ratio and P(A-a)O_2_. Additional ways in which the relationship between ventilation and blood oxygenation could be impaired include shunting, but we were unable to measure shunt fractions, or regional ventilation perfusion relationships in the lungs.

Etorphine not only had cardiovascular effects on the pulmonary circulation. Although we did not measure right atrial pressure, and therefore could not calculate systemic vascular resistance, we do know that, after etorphine administration, mean systemic arterial pressure dropped at a time at which cardiac output had not changed. The likely explanation was that etorphine’s effects induced systemic vasodilation, in contrast to the pulmonary vasoconstriction that it induced. The modest increase in pulmonary artery wedge pressure, implying a modest increase in left atrial pressure, and the eventual decline in cardiac output, presumably indicate an effect of etorphine on the heart itself. We note, too, that there was no immediate change in heart rate following etorphine administration in spite of changes in systemic arterial pressure, possibly reflecting etorphine-induced impairment of the baroreceptor reflex.

We are not the first to report that etorphine, and other potent opioids like carfentanil, cause pulmonary hypertension [15], a response that has been implicated in the development of severe pulmonary oedema in animals [18,19]. Pulmonary hypertension and oedema also occur in humans who abuse heroin, morphine, opium and methadone [20]. As far as we are aware, the mechanisms by which acute opioid administration influences pulmonary oxygen exchange have not been explored previously. The effects of pulmonary hypertension on gas exchange have been studied in chronic pulmonary disease [21] and heart failure [22,23], where pulmonary hypertension can either have a protective or pathophysiological effects. Not all studies on the effects of opioids on the pulmonary vasculature are congruent; in the pulmonary vasculature of cats, Hakim *et al.* [20] found that morphine had vasoconstrictor effects whereas Kaye *et al.* [24] found vasodilator effects. The reasons for this disparity are not clear, but, intriguingly, in both studies the vasoactive effects described were antagonised by naloxone and chlorpheniramine or diphenhydramine, indicating that these effects were mediated or modulated by both opioid-receptor and histamine-receptor sensitive pathways [20,24].

In our study, acute etorphine administration led to pulmonary hypertension and systemic hypotension. The increased pulmonary artery pressures had a significant and important clinical impact on our goats. Traditionally hypoxia following etorphine administration has been attributed to its depressive effects on respiratory rhythmogenesis and control (i.e. opioid-induced respiratory depression). However, we have shown, for the first time, that the initial and most severe hypoxia caused by etorphine results mainly from the effects of pulmonary hypertension on alveoli gas exchange. Although these effects were temporary in our goats, their magnitude and timing is clinically significant as the first ten minutes after opioid administration is the time when the most lethal complications from etorphine immobilization can occur [4]. Whether similar pulmonary hypertension, causing a decrease in pulmonary gas exchange, results from administration of other opioids still needs to be investigated, particularly for those opioids used as immobilizing and anaesthetic induction agents.

## Conclusion

Because etorphine-induced pulmonary hypertension played such an important role in the development and severity of hypoxia, methods of alleviating respiratory derangements from etorphine, and possibly other opioid agonists, should focus not only on reversing the opioid-induced central respiratory depression, but also on improving oxygen movement across the alveoli membrane, by preventing pulmonary vasoconstriction particular in areas of the lungs that are well ventilated.
